# The Temporal Lag Structure of Short-term Associations of Fine Particulate Matter Chemical Constituents and Cardiovascular and Respiratory Hospitalizations

**DOI:** 10.1289/ehp.1104721

**Published:** 2012-05-18

**Authors:** Sun-Young Kim, Jennifer L Peel, Michael P Hannigan, Steven J Dutton, Lianne Sheppard, Maggie L Clark, Sverre Vedal

**Affiliations:** 1 Department of Environmental and Occupational Health Sciences, University of Washington, Seattle, Washington, USA; 2 Department of Environmental and Radiological Health Sciences, Colorado State University, Fort Collins, Colorado, USA; 3 Department of Mechanical Engineering, University of Colorado, Boulder, Colorado, USA; 4 National Center for Environmental Assessment, U.S. Environmental Protection Agency, Research Triangle Park, North Carolina, USA; 5 Department of Biostatistics, University of Washington, Seattle, Washington, USA

**Keywords:** air pollution, cardiovascular disease, chemical constituent, hospital admission, particulate matter, respiratory disease, time-series study

## Abstract

Background: In air pollution time-series studies, the temporal pattern of the association of fine particulate matter (PM_2.5_; particulate matter ≤ 2.5 µm in aerodynamic diameter) and health end points has been observed to vary by disease category. The lag pattern of PM_2.5_ chemical constituents has not been well investigated, largely because daily data have not been available.

Objectives: We explored the lag structure for hospital admissions using daily PM_2.5_ chemical constituent data for 5 years in the Denver Aerosol Sources and Health (DASH) study.

Methods: We measured PM_2.5_ constituents, including elemental carbon, organic carbon, sulfate, and nitrate, at a central residential site from 2003 through 2007 and linked these daily pollution data to daily hospital admission counts in the five-county Denver metropolitan area. Total hospital admissions and subcategories of respiratory and cardiovascular admissions were examined. We assessed the lag structure of relative risks (RRs) of hospital admissions for PM_2.5_ and four constituents on the same day and from 1 to 14 previous days from a constrained distributed lag model; we adjusted for temperature, humidity, longer-term temporal trends, and day of week using a generalized additive model.

Results: RRs were generally larger at shorter lags for total cardiovascular admissions but at longer lags for total respiratory admissions. The delayed lag pattern was particularly prominent for asthma. Elemental and organic carbon generally showed more immediate patterns, whereas sulfate and nitrate showed delayed patterns.

Conclusion: In general, PM_2.5_ chemical constituents were found to have more immediate estimated effects on cardiovascular diseases and more delayed estimated effects on respiratory diseases, depending somewhat on the constituent.

In time-series air pollution studies, it has been common practice to explore the temporal pattern of associations between daily counts of health end points and daily ambient concentrations of fine particulate matter (PM_2.5_; particulate matter ≤ 2.5 µm in aerodynamic diameter) on the same and a few previous days (Dominici et al. 2006; Lippmann et al. 2000; Samet et al. 2000; Wong et al. 2010). Different patterns of lag effects for different disease outcomes may provide insight into different biological mechanisms of acute responses to particulate matter air pollution (Zanobetti et al. 2003). Reported temporal patterns of the associations of PM_2.5_ have differed for cardiovascular and respiratory diseases. Specifically, hospital admissions for cardiovascular diseases, such as heart failure, cerebrovascular disease, and abnormal heart rhythm, and emergency department visits and hospital admissions for ischemic stroke and transient ischemic attack have tended to show greater PM_2.5_ health effect estimates on the same day or the previous exposure day (Dominici et al. 2006; Ito et al. 2011; Lisabeth et al. 2008; Metzger et al. 2004). In contrast, emergency department visits for asthma and hospital admissions for respiratory tract infection showed larger health effect estimates for lags of 2–6 days (Dominici et al. 2006; Peel et al. 2005).

Recently, there has been growing interest in studying short-term effects of chemical constituents of PM_2.5_ for the purpose of identifying the most toxic chemical constituents of PM_2.5_ (Ito et al. 2011; Lall et al. 2011; Ostro et al. 2009; Peng et al. 2009; Zhou et al. 2011). Lag patterns of PM_2.5_ constituents, acting as indicators of pollution sources, may provide new insights into the underlying relationships between PM_2.5_ air pollution and health. However, it has been difficult to investigate the daily lag structure of the association between particulate matter constituents and health outcomes because most U.S. studies have relied on U.S. Environmental Protection Agency (EPA) regulatory monitoring data derived from networks using every third or sixth day sampling schedules. In the Denver Aerosol Sources and Health (DASH) study, which was designed to assess associations between short-term exposure to PM_2.5_ chemical constituents and sources with health outcomes, PM_2.5_ constituents were measured daily for 5 years, thereby overcoming this limitation (Vedal et al. 2009). In the present study, we used daily speciated PM_2.5_ data in Denver, Colorado, from 2003 through 2007 to explore the lag structure of the association between PM_2.5_ constituents and hospital admissions by disease.

## Methods

**Figure 1:**
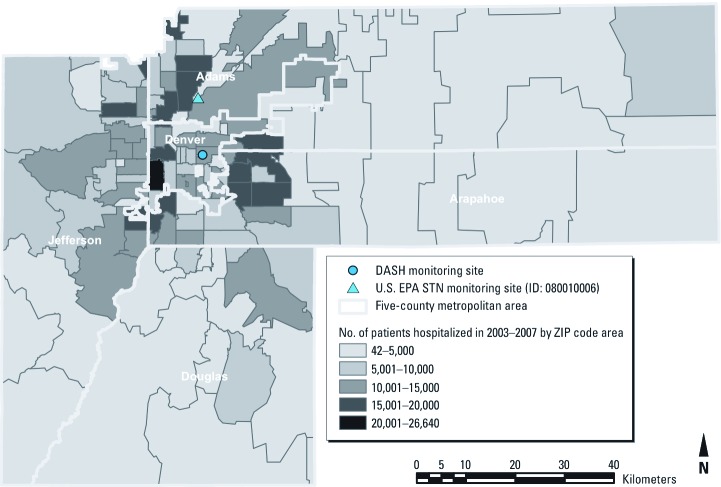
Map showing the DASH monitoring site within the five-county Denver metropolitan area and total hospital admissions by ZIP code for 2003 through 2007. STN, Speciation Trends Network; ID, U.S. EPA database monitoring identification code.

PM_2.5_ mass and chemical constituents were measured daily at one residential monitoring station located on the roof of an elementary school building in Denver from 2003 through 2007 (Vedal et al. 2009). This monitoring site was selected to represent residential concentrations of PM_2.5_ constituents in Denver and to not be influenced by nearby pollution emission sources, including large roadways (Figure 1). Sampling and analysis protocols of PM_2.5_ constituents have been described in detail elsewhere (Dutton et al. 2009; Vedal et al. 2009). Here, we focus on four PM_2.5_ chemical constituents that comprise the majority of PM_2.5_ mass in Denver: elemental carbon (EC), organic carbon (OC), sulfate, and nitrate (Vedal et al. 2009). EC and OC were collected on quartz fiber filters and analyzed using thermal optical transmission; sulfate and nitrate were collected on Teflon filters and analyzed using ion chromatography (Dutton et al. 2009).

All individual hospital admission records during the study period were extracted from nonelective hospital admission discharge data obtained from the Colorado Hospital Association (2012). We used daily counts of hospital admissions for hospitals located within the five county Denver metropolitan area (Adams, Arapahoe, Denver, Douglas, and Jefferson counties); admissions were restricted to patients who resided in these same five counties. About 90% of the five-county population lived within 25 km of our monitoring site. We selected non-elective hospital admissions, including those classified as emergency, urgent, or trauma admissions. The *International Classification of Diseases, Ninth Revision* (ICD-9) codes were used to define cardiovascular hospital admissions (codes 390–459) and respiratory hospital admissions (codes 460–519) (World Health Organization 1978). Subgroups of cardiovascular hospital admissions were categorized as ischemic heart disease (ICD-9 codes 410–414), congestive heart failure (ICD-9 code 428), and cerebrovascular disease (ICD-9 codes 430–438). Respiratory hospital admissions were classified as chronic obstructive pulmonary disease (COPD; ICD-9 codes 490–492, 496), asthma (ICD-9 code 493), and pneumonia (ICD-9 codes 480–486). The DASH study has maintained approval by the University of Washington and Colorado State University institutional review boards.

We estimated the association of 24-hr daily average concentration of each PM_2.5_ constituent and daily count of hospital admissions using a generalized additive model, after adjusting for days from the start of the study, day of the week, and 24-hr daily average temperature and 24-hr daily average relative humidity. We accounted for nonlinear temporal and temperature trends using regression splines with the degrees of freedom (df) for each term selected *a priori*: 12 df per year for time and 3 df for temperature (Wood 2006). We used same-day weather covariates because in the sensitivity analyses, temperature and humidity on single or multiple lag days did not alter the results. We present relative risks (RRs) and 95% confidence intervals (CIs) for an interquartile range (IQR) increase in PM_2.5_ constituents. Lagged patterns were assessed by disease category for the PM_2.5_ constituent concentration on the current day and each of 14 days before hospital admission using a constrained distributed lag model. Specifically, the constrained lag model was based on natural cubic B-splines with 4 df; this choice gave the smallest Akaike information criterion across the four constituents. In the sensitivity analyses, we also estimated lag patterns using fifteen single-day lag models, an unconstrained distributed lag model, and different parameterizations of constrained distributed lag models such as polynomial and polynomial spline models with different degrees of freedom. In addition, we examined the sensitivity of the lag pattern to adjustment for gaseous copollutants, including carbon monoxide, sulfur dioxide, nitrogen dioxide, and ozone, to restriction of the study area to Denver County, and to the use of fewer degrees of freedom (4 df and 6 df/year) for time.

## Results

In the five-county Denver metropolitan area, average daily mean concentrations of PM_2.5_ mass, EC, OC, sulfate, and nitrate from 2003 through 2007 were 7.98, 0.47, 3.09, 1.08, and 1.03 ug/m^3^, respectively (Table 1). EC and OC showed strong seasonal trends with concentration peaks in the winter for EC, and in both summer and winter for OC, whereas both constituents were lowest in the spring (not shown; see Dutton et al. 2010a). Daily sulfate and nitrate concentrations were more highly correlated with PM_2.5_ mass concentration than were EC and OC (Table 2). Seasonal trends for all four constituents were similar to those at the U.S. EPA Speciation Trends Network (STN) site located about thirteen kilometers north of the DASH study monitoring site (not shown; see Vedal et al. 2009).

**Table 1:** Summary statistics for 24-hr averages of PM_2.5_ total and component mass and meteorology and for daily counts of hospitalization for 2003 through 2007 in the five-county Denver metropolitan area.

Category/variable	No. of days	Minimum	Median	Maximum	IQR	Mean ± SD
Pollutants (µg/m3)												
PM2.5		1,808		–0.92a		6.87		59.41		4.54		7.98 ± 5.08
EC		1,809		0.00		0.40		3.02		0.33		0.47 ± 0.33
OC		1,809		–0.78a		2.91		10.28		1.67		3.09 ± 1.39
Sulfate		1,808		0.00		0.91		14.32		0.76		1.08 ± 0.97
Nitrate		1,808		–0.02a		0.22		19.72		0.86		1.03 ± 1.97
Meteorology												
Temperature (°F)		1,813		–4.6		51.5		85.4		27.8		51.1 ± 17.4
Humidity (%)		1,813		14.3		51.8		100.0		30.5		54.6 ± 20.8
Primary diagnosis for hospital admission												
Cardiovascular disease		1,826		20		44.5		75		14		44.8 ± 9.3
Ischemic heart disease		1,826		0		11		29		5		11.7 ± 4.0
Congestive heart failure		1,826		0		8		23		5		7.8 ± 3.2
Cerebrovascular disease		1,826		0		7		20		3		7.4 ± 2.8
Respiratory disease		1,826		5		34		107		18		37.1 ± 14.6
Chronic Obstructive Pulmonary Disease		1,826		0		5		20		3		4.9 ± 2.7
Asthma		1,826		0		6		22		4		5.8 ± 3.0
Pneumonia		1,826		2		12		37		7		12.8 ± 5.6
aNegative values resulted from the uncertainty of some measurements being as large as the measurements themselves (Dutton et al. 2010a).												

**Table 2:** Pearson correlation coefficients between 24-hr PM_2.5_ mass and components and meteorological conditions for 2003 through 2007 in the five-county Denver metropolitan area.

Pollutant	PM_2.5_	EC	OC	Sulfate	Nitrate	Temperature
EC		0.46										
OC		0.54		0.55								
Sulfate		0.68		0.09		0.20						
Nitrate		0.82		0.36		0.26		0.56				
Temperature		–0.23		–0.20		0.18		–0.05		–0.53		
Humidity		0.34		0.02		–0.14		0.36		0.47		–0.52

**Figure 2:**
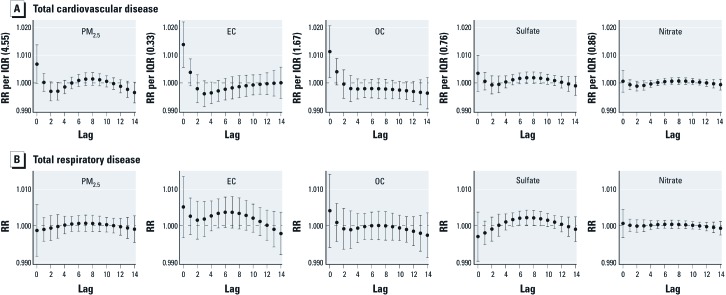
The pattern of RRs from lag 0 to lag 14 estimated from a constrained distributed lag model of total cardiovascular disease and respiratory disease hospitalizations for an IQR increase in PM_2.5_ mass and the four chemical components using data from 2003 through 2007 within the five-county Denver metropolitan area.

**Figure 3:**
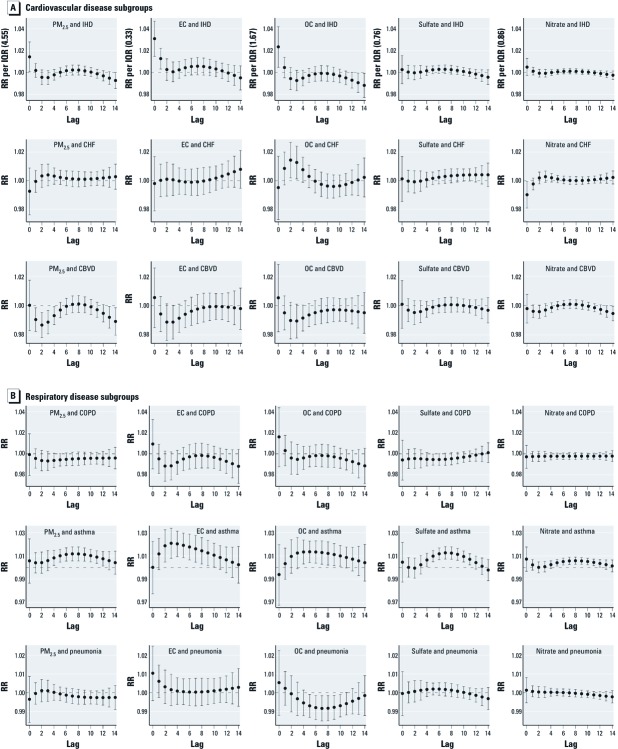
The pattern of RRs from lag 0 to lag 14 estimated from a constrained distributed lag model of cause-specific hospitalizations for cardiovascular diseases [ischemic heart disease (IHD), congestive heart failure (CHF), cerebrovascular disease (CBVD), and ischemic stroke (IS)] and for respiratory diseases (COPD, asthma, and pneumonia) for an IQR increase in PM_2.5_ and the four chemical components. Data from 2003 through 2007 and the five-county Denver metropolitan area.

On average, 236 people were hospitalized each day. Of these admissions, 45 (19%) had a discharge diagnosis of a cardiovascular disease, and 37 (16%) had a discharge diagnosis of a respiratory disease each day on average (Table 1). Forty-four percent of all hospitalized patients were > 65 years of age, and 9% were < 19 years of age. Figure 2 shows the health effect estimates from the constrained distributed lag models for PM_2.5_ mass and the four constituents by disease category. The largest effects of constituents on cardiovascular hospital admissions were generally estimated at shorter pollutant lags, whereas larger effect estimates for respiratory hospital admissions tended to occur at longer lags. Specifically, RRs for IQR increases in PM_2.5_ mass, EC, and OC were highest at lag 0 and decreased sharply afterwards for total cardiovascular hospital admissions, whereas total respiratory hospital admissions did not have dramatically elevated RRs at lag 0 or at lag 1. Among the various subcategories of cardiovascular hospital admissions, ischemic heart disease showed dominant immediate effects for EC and OC that paralleled the pattern of total cardiovascular disease admissions (Figure 3). Among the respiratory outcomes, hospital admission for asthma showed the most obvious delayed lag effects, with elevated effects beginning at lag 2–5, depending upon the constituent. Sulfate and nitrate tended to show more delayed patterns than did EC and OC. We observed no statistically significant increases in RR at any lags in hospital admissions for cerebrovascular disease, COPD, or pneumonia.

Total cumulative effects were also estimated from the constrained distributed lag models for 14 days. When pollutant levels on the previous 2–14 days were adjusted, the estimated cumulative RRs of cardiovascular disease hospital admissions over lag 0 to lag 1 for interquartile increases in PM_2.5_, EC, OC, sulfate, and nitrate were 1.007 (95% CI: 0.998, 1.016), 1.018 (95% CI: 1.006, 1.030), 1.015 (95% CI: 1.002, 1.029), 1.004 (95% CI: 0.995, 1.013), and 1.000 (95% CI: 0.995, 1.005), respectively. Estimated cumulative RRs of respiratory disease hospital admissions over lag 0 to lag 14 were 0.998 (95% CI: 0.974, 1.024), 1.032 (95% CI: 0.983, 1.082), 0.994 (95% CI: 0.953, 1.038), 1.007 (95% CI: 0.985, 1.029), and 1.001 (95% CI: 0.989, 1.014), respectively.

In the sensitivity analyses that compared different modeling approaches for assessing lag structure, we found the pattern of cardiovascular and respiratory diseases hospital admissions for EC, as an example, was reasonably similar across approaches, although, not surprisingly, effect estimates from the unconstrained distributed lag model and the fifteen single-day lag models were less stable and had wider confidence limits than did the estimates from the constrained distributed lag models [see Supplemental Material, Figure S1 (http://dx.doi.org/10.1289/ehp.1104721)]. A similar conclusion was reached from assessment of findings using these several alternative models with the other PM_2.5_ constituents (results not shown). The observed lag patterns were consistent with the primary results when the models included terms for daily concentrations of each of four gaseous pollutants on the same day (results not shown) (see Supplemental Material, Tables S1 and S2 for summary statistics and correlations of gaseous pollutants). The restriction of the study area and the use of fewer degrees of freedom for time did not meaningfully alter the patterns (results not shown).

## Discussion

This study explored the temporal lag patterns of the effects of PM_2.5_ chemical constituent concentrations by disease category on hospital admissions in the Denver metropolitan area. We generally found stronger associations of cardiovascular hospital admissions and PM_2.5_ constituents on the same day, whereas respiratory admissions had stronger associations with PM_2.5_ constituent concentrations after a delay (lag) of a few days. At short time lags, the estimated effects of EC and OC were larger than were those of sulfate and nitrate.

To our knowledge, very few time-series studies have been able to explore the extended lag structure of short-term exposure to PM_2.5_ chemical constituents using daily concentration measurements. Most time-series studies of PM_2.5_ chemical constituents carried out in the United States have used regulatory monitoring data from the U.S. EPA Chemical Speciation Network (CSN) (Ito et al. 2011; Ostro et al. 2009; Peng et al. 2009). Within the CSN, 53 core sites (recognized as STN sites) sample every third day, whereas approximately 197 supplementary sites generally sample every sixth day (U.S. EPA 2009). With such temporally sparse exposure data, previous studies by necessity have typically estimated effects either at lag 0 or at lag 3 for a given event day (Ostro et al. 2009) or at several consecutive lags, but using different event days (Ito et al. 2011; Peng et al. 2009). This results in not only limited statistical power, but in an inability to compare effects of different lags on the outcome for the same event day. The sampling limitations of CSN monitoring data have been identified as a challenge in exploring lag structure of PM_2.5_ constituents that could be addressed in future studies with more temporally rich monitoring data (Lippmann 2009; Ostro et al. 2007). Some studies performed in Atlanta, Georgia, and New York City have employed daily PM_2.5_ constituent measurements (Lall et al. 2011; Metzger et al. 2004; Peel et al. 2005), but for relatively short sampling periods of approximately 2 years (and for different outcome data such as Medicare hospital admissions or emergency department visits). The present study benefits from the increased power obtained by having rich daily PM_2.5_ constituent concentration data conducted over a period of 5 years.

Consistent with our results, previous studies have suggested that there are different lag structures for cardiovascular and respiratory diseases. Ostro et al. (2009) examined the relationship between PM_2.5_ constituents and respiratory hospital admissions among children who resided in California and found stronger effect estimates of EC at lag 3 than at lag 0. Peel et al. (2005) reported stronger effect estimates at longer lags (lag 4 and lag 11) for asthma emergency department visits for EC in Atlanta, Georgia. In contrast, estimated effects were elevated on the same day (lag 0) for EC for cardiovascular emergency department visits in the same study (Metzger et al. 2004). Peng et al. (2009) compared cardiovascular and respiratory hospital admissions using Medicare data and found relative risks for EC were larger at lag 0 for cardiovascular hospital admissions and at lag 2 for respiratory hospital admissions. Lall et al. (2011) also reported larger effect estimates of EC on cardiovascular hospital admissions at lag 0 among New York Medicare patients, but at lag 3 for respiratory hospital admissions. Taking advantage of 5 years of daily speciation data, we confirmed these previous findings of the patterns for EC and OC by showing that cardiovascular and respiratory diseases exhibited different patterns.

It is not yet clear why temporal associations vary by disease groups. It is plausible that cardiac responses occur immediately following autonomic nervous system activation, whereas respiratory responses may take longer because of disease exacerbation resulting from inflammation (Brook et al. 2010; Ostro et al. 2009). In addition to differences in biologic mechanism, the different lag structures could be due to patient behavior patterns that differ by disease. Cardiovascular diseases such as myocardial infarction and stroke could prompt more immediate hospital admission, whereas it may take time for respiratory conditions to be considered sufficiently severe to warrant hospitalization. Also, in the United States, the elderly, who make up the majority of those with cardiovascular disease admissions, may be more likely to visit hospitals relatively shortly after the onset of symptoms given reduced financial barrier to medical care access as Medicare beneficiaries. Future studies are needed to clarify the causes of the differences we observed.

The patterns of temporal lag structure of hospital admissions varied by PM_2.5_ chemical constituents, although overall the contrast between cardiovascular and respiratory disease outcomes was similar across constituents. EC and OC showed more obvious immediate effects than did sulfate and nitrate. The immediate lag effects estimated for cardiovascular diseases were most distinct for EC and OC. Delayed patterns of increased RRs for asthma began at later lags for sulfate and nitrate than for EC and OC. EC and OC are of particular interest as markers of combustion. In particular, EC is often considered a marker of diesel exhaust, although other combustion sources such as wood burning also contribute to EC [Health Effects Institute (HEI) 2010; Schauer 2003]. Source-apportioned particulate matter in the Denver area has shown very little contribution from wood smoke for data collected in 1996 and 1997 (Watson et al. 1998) as well as in 2003 (Dutton et al. 2010b). The different pattern of EC concentrations between weekdays and weekends found in the DASH study combined with vehicle traffic count data also suggest that diesel exhaust is the main contributor to EC in Denver (Dutton et al. 2010a). Different lag patterns of the carbon fractions (EC and OC) and the secondary pollutants (sulfate and nitrate) may reflect effects of different sources or processes in the Denver area, and of the combination of pollutants that is produced by these source or processes. Future work utilizing source apportionment on an expanded set of PM_2.5_ constituents will examine whether the observed lag structures of PM_2.5_ constituents correspond to or differ from those derived from individual PM_2.5_ sources (Dutton et al. 2010b).

We chose a constrained distributed lag model based on natural cubic B-splines as our primary approach to displaying the pattern of lags across days. As another approach in time-series health studies, cumulative-day effects have often been presented aggregated over incrementally longer periods of time to assess temporal lag structures using the unconstrained or constrained distributed lag models (Schwartz 2000; Zanobetti et al. 2002, 2003). Our decision to display single-day lag effects instead of cumulative-day effects was based on our interest in explicitly illustrating the day-to-day lag patterns. In addition, the constrained distributed lag model allowed us to gain better insight into the shape of temporal relationship by reducing noise (Schwartz 2000). However, each type of constraint imposes some structure on the results; ideally this choice is based on prior scientific knowledge about the pattern of effects (Welty et al. 2009). Since we did not have a firm prior hypothesis, we explored the sensitivity of our model by comparing the lag patterns from our natural cubic spline models to those from fifteen single-day lag models, unconstrained distributed lag models, and two other constrained distributed lag models. Although single-day lag models and unconstrained distributed lag models gave less stable estimates than those from constrained distributed lag models, our main findings were supported by generally consistent patterns across models [see Supplemental Material, Figure S1 (http://dx.doi.org/10.1289/ehp.1104721)].

It should be noted that we mostly relied on informal tests to report different lag patterns by diseases and constituents. For cardiovascular diseases, for EC and OC, the constrained lag models estimated by natural cubic splines gave significantly better fit based on the likelihood ratio test than the constrained lag models with one parameter that assume no patterns. However, for respiratory diseases and all constituents, there was no evidence of statistically significant improvement in model fit when lag effects were constrained using natural splines, possibly because of the gradual increase and decrease in patterns of associations for respiratory diseases, in contrast with the dramatic early increase in patterns seen for cardiovascular diseases with EC and OC. For assessing the difference of lag patterns between the diseases and between constituents, we relied on visual inspections only.

Initial exploratory analyses (results not shown) suggested that the elderly > 65 years of age had more immediate associations with PM_2.5_ constituents for hospital admissions and children < 19 years of age had more delayed associations. This age difference likely is due at least in part to the different age distributions of cardiovascular and respiratory disease hospitalizations. Respiratory hospital admissions made up 43.6% of children’s hospital admissions, but only 15.2% of hospital admissions among the elderly. In contrast, cardiovascular hospital admissions made up only 1.4% of children’s hospital admissions, but 26.3% of those in the elderly.

One of the limitations in this study was our reliance on only one residential monitoring site for our ambient PM_2.5_ measurements. This single site is less of a concern in our study since most of the study population lived relatively close to the monitoring site. Furthermore, the correlations between our monitor and the nearby STN monitor (about 13 km away) were fairly strong: 0.55, 0.71, 0.61, 0.8, and 0.85 for PM_2.5_, EC, OC, sulfate, and nitrate, respectively. Because these two sites had also similar shapes of their time trends over the 5 years for all four constituents, we were reassured that our assumption of a reasonably homogenous distribution of constituents over space was valid (Vedal et al. 2009). This suggests less bias in the health effect estimates when using a time-series study design (Zeger et al. 2000; Sarnat et al. 2010). Although we considered the use of measurements from the STN site, because of the spatial homogeneity, its siting in an industrial area, and most importantly, its one-in-three day monitoring schedule, we decided not to include this location. However, our comparison to only one other site is limited and may not provide an adequate assessment. Constituent concentrations might vary over local spatial scales that are not captured by these monitors and thereby contribute to exposure measurement error. For example, EC is considered to be largely affected by local sources such as traffic (HEI 2010); there is therefore likely to be considerable heterogeneity at small spatial scales, not reflected in the comparison of the DASH site and the neighboring STN site. Others have reported that the distribution of PM_2.5_ chemical constituents over space is not homogeneous (Bell et al. 2007, 2011). In order to better understand spatial heterogeneity of PM_2.5_ constituents in Denver, we have carried out supplemental monitoring at three additional sites for 1 year and plan to compare the PM_2.5_ constituent time series across these multiple monitoring sites.

## Conclusions

In summary, we observed that estimated short-term effects of PM_2.5_ chemical constituents, especially those of EC and OC, were more immediate for cardiovascular diseases and more delayed for respiratory diseases. Future work will determine whether specific sources of PM_2.5_ exhibit similar temporal patterns of effects and how spatial heterogeneity influences the results.

## Supplemental Material

(61 KB) PDF
