# Supplementary material for: The Temporal Lag Structure of Short-term Associations of Fine Particulate Matter Chemical Constituents and Cardiovascular and Respiratory Hospitalizations
Source: Environ Health Perspect. 2012 May 18;120(8):1094–9. doi: 10.1289/ehp.1104721 (PMC3440088; doi:10.1289/ehp.1104721)
Supplement: (61 KB) PDF [file ehp.1104721.s001.pdf]

Supplemental Material

The Temporal Lag Structure of Short-term Associations of Fine Particulate Matter Chemical Constituents and Cardiovascular and Respiratory Hospitalizations

Sun-Young Kim[1], Jennifer L. Peel[2], Michael P. Hannigan[3], Steven J. Dutton[4], Lianne Sheppard[1,5], Maggie L. Clark[2], Sverre Vedal[1]

[1]Department of Environmental and Occupational Health Sciences, University of Washington, Seattle, WA, USA

[2]Department of Environmental and Radiological Health Sciences, Colorado State University, Fort Collins, CO, USA

[3]Department of Mechanical Engineering, University of Colorado, Boulder, CO, USA

[4]U.S. Environmental Protection Agency, National Center for Environmental Assessment, RTP, NC, USA

[5]Department of Biostatistics, University of Washington, Seattle, WA, USA

Table of Contents

Table S1. Summary statistics for gaseous pollutant concentrations for 2003-2007 in the five-county Denver metropolitan area.

Table S2. Pearson correlation coefficients between daily $PM_{2.5}$ mass and components and gaseous pollutants for 2003-2007 in the five-county Denver metropolitan area.

Figure S1. The pattern of relative risks (RRs) from lag 0 to lag 14 estimated from three constrained distributed lag models (natural cubic B-spline, polynomial B-spline, and polynomial models), an unconstrained distributed lag model, and fifteen separate single-day lag models for cardiovascular disease (CVD on the top) and respiratory disease (RD on the bottom) hospitalizations for an interquartile range increase in EC. Data from 2003-2007 and the five-county Denver metropolitan area.

Supplemental Material Table S1. Summary statistics for gaseous pollutant concentrations for 2003-2007 in the five-county Denver metropolitan area.

Pollutant (ppm)	N	Min	Q1	Median	Q3	Max	IQR	Mean	SD
CO*	1779	0.40	0.90	1.20	1.70	15.00	0.80	1.48	0.97
SO_2*	1675	0.00	0.01	0.01	0.01	0.06	0.01	0.01	0.01
NO_2*	1578	0.01	0.04	0.05	0.06	0.14	0.02	0.05	0.01
Ozone**	1729	0.00	0.03	0.04	0.05	0.11	0.02	0.04	0.02

*Maximum of daily 1-hour averages

**Maximum of daily 8 hour averages

Supplemental Material Table S2. Pearson correlation coefficients between daily $PM_{2.5}$ mass and components and gaseous pollutants for 2003-2007 in the five-county Denver metropolitan area.

Pollutant	Mass	EC	OC	Sulfate	Nitrate	CO	SO_2	NO_2
CO*	0.23	0.63	0.30	-0.03	0.20			
SO_2*	0.23	0.35	0.31	0.01	0.14	0.34		
NO_2*	0.26	0.37	0.32	-0.01	0.16	0.50	0.34	
Ozone**	-0.30	-0.47	0.00	-0.06	-0.49	-0.46	-0.13	-0.14

*Maximum of daily 1-hour averages

**Maximum of daily 8 hour averages

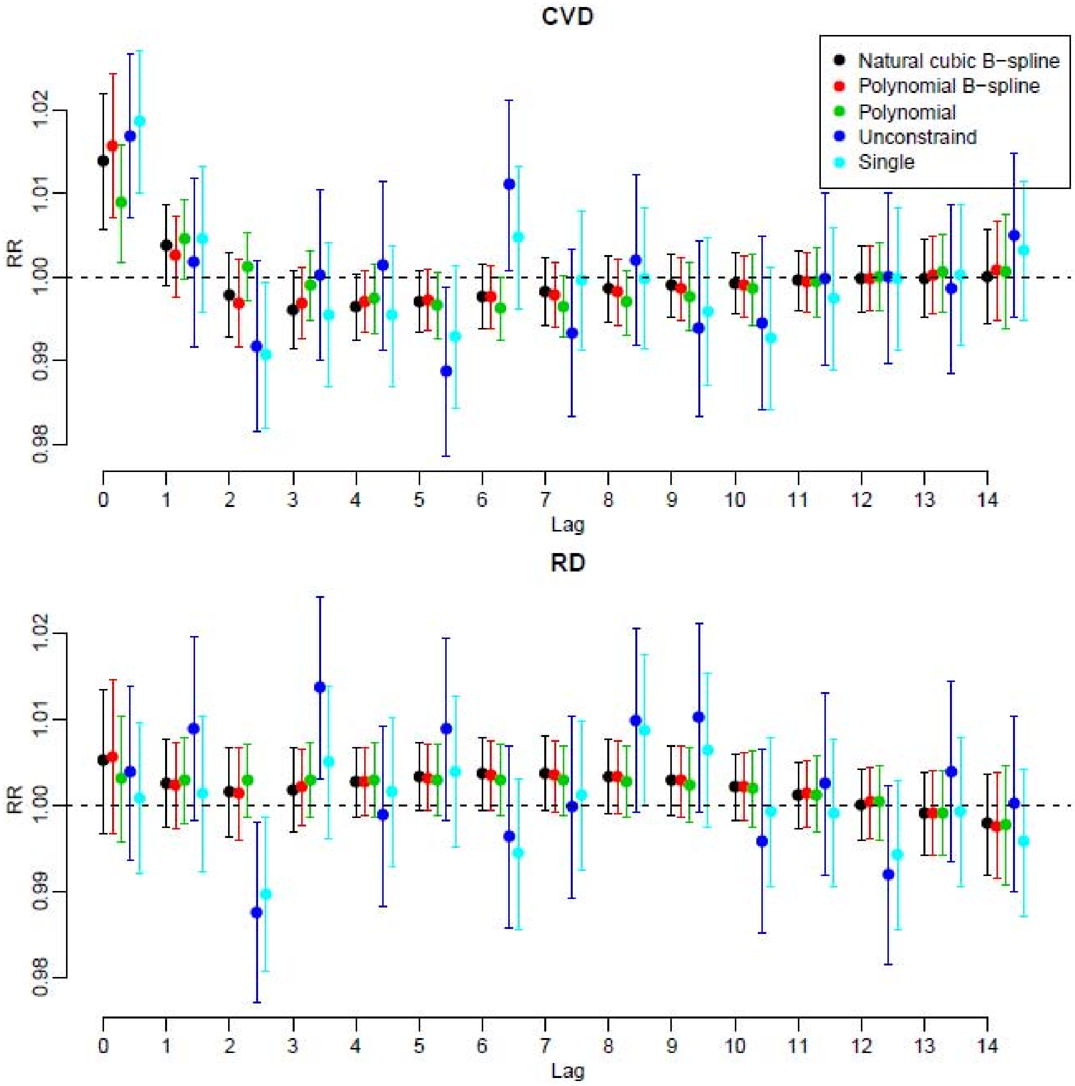

Supplemental Material Figure S1. The pattern of relative risks (RRs) from lag 0 to lag 14 estimated from three constrained distributed lag models (natural cubic B-spline, polynomial B-spline, and polynomial models), an unconstrained distributed lag model, and fifteen separate single-day lag models for cardiovascular disease (CVD on the top) and respiratory disease (RD on the bottom) hospitalizations for an interquartile range increase in EC. Data from 2003-2007 and the five-county Denver metropolitan area.
